# Elevated Serum Levels of the Antiapoptotic Protein Decoy-Receptor 3 Are Associated with Advanced Liver Disease

**DOI:** 10.1155/2016/2637010

**Published:** 2016-08-09

**Authors:** Giorgos Bamias, Michalis Gizis, Ioanna Delladetsima, Eyfrosyni Laoudi, Spyros I. Siakavellas, Ioannis Koutsounas, Garyfallia Kaltsa, John Vlachogiannakos, Irene Vafiadis-Zouboulis, George L. Daikos, George V. Papatheodoridis, Spiros D. Ladas

**Affiliations:** ^1^Academic Department of Gastroenterology, Medical School of National and Kapodistrian University of Athens, Laiko General Hospital, Athens 11527, Greece; ^2^First Department of Pathology, Medical School of National and Kapodistrian University of Athens, Laiko General Hospital, Athens 11527, Greece; ^3^Infectious Diseases Research Laboratory, Medical School of National and Kapodistrian University of Athens, Laiko General Hospital, Athens 11527, Greece

## Abstract

*Background*. Decoy-receptor 3 (DcR3) exerts antiapoptotic and immunomodulatory function and is overexpressed in neoplastic and inflammatory conditions. Serum DcR3 (sDcR3) levels during the chronic hepatitis/cirrhosis/hepatocellular carcinoma (HCC) sequence have not been explored.* Objective*. To assess the levels and significance of sDcR3 protein in various stages of chronic liver disease.* Methods*. We compared sDcR3 levels between healthy controls and patients with chronic viral hepatitis (CVH), decompensated cirrhosis (DC), and HCC. Correlations between sDcR3 levels and various patient- and disease-related factors were analyzed.* Results*. sDcR3 levels were significantly higher in patients with CVH than in controls (*P* < 0.01). sDcR3 levels were elevated in DC and HCC, being significantly higher compared not only to controls (*P* < 0.001 for both) but to CVH patients as well (*P* < 0.001 for both). In addition, DcR3 protein was detected in large quantities in the ascitic fluid of cirrhotics. In patients with CVH, sDcR3 significantly correlated to fibrosis severity, as estimated by Ishak score (*P* = 0.019) or by liver stiffness measured with elastography (Spearman *r* = 0.698, *P* < 0.001). In cirrhotic patients, significant positive correlations were observed between sDcR3 levels and markers of severity of hepatic impairment, including MELD score (*r* = 0.653, *P* < 0.001).* Conclusions*. Circulating levels of DcR3 are elevated during chronic liver disease and correlate with severity of liver damage. sDcR3 may serve as marker for liver fibrosis severity and progression to end-stage liver disease.

## 1. Introduction

Hepatic carcinogenesis is a multistep process, which develops through sequential stages [1]. Hepatocellular carcinoma (HCC) develops mainly on cirrhotic livers through malignant transformation of regenerative nodules. Cirrhosis is usually the end result of chronic necroinflammatory activity (chronic hepatitis) which causes repeated cycles of hepatic injury and repair [2]. The critical biological event is the development of fibrosis, which eventually leads to distortion of liver architecture and deterioration of hepatic function. Discrimination between different stages is of great importance for the individual patient, as prognostic significance and therapeutic implications usually differ significantly in each stage. Therefore, the identification of markers which can be easily detected and are associated with the severity of fibrosis would be of great clinical application.

Decoy-receptor 3 (DcR3) belongs to the tumor necrosis factor receptor superfamily of proteins (TNFRSF). It is capable of binding to three different TNF-like ligands, FasL, TL1A, and LIGHT, thus blocking their interaction with their functional receptors (Fas, DR3, and LTR*β*, resp.) [3, 4]. Through this inhibitory function, DcR3 exerts antiapoptotic and immunomodulatory properties. Accordingly, it was proposed that DcR3 may promote tumorigenesis not only by directly inhibiting tumor cell apoptosis but also by ameliorating cytolytic T-cell responses and recruitment of acute inflammatory cells, as well as by promoting angiogenesis. In addition, DcR3 has also been shown to block proinflammatory signals mediated by the aforementioned TNF/TNFRSF ligand-receptor pairs. In line with these functional data, expression studies have clearly demonstrated overexpression of DcR3 in a variety of inflammatory and neoplastic conditions [5–8]. A distinctive property of DcR3 is that it lacks a transmembrane region and exists only as soluble protein, which can be accurately determined in biological fluids [9]. DcR3 is not detected or exists in low levels in the peripheral blood of healthy individuals; but it is secreted in the systemic circulation, often reaching substantial concentrations, during several inflammatory and neoplastic conditions [7, 10–13].

The dual biological functionality of DcR3 (i.e., immunoregulatory and antiapoptotic) raises the possibility that it may be involved in the chronic hepatitis/cirrhosis/HCC sequence, as inflammation takes place in the initial and malignant transformation in the late stage. Previous studies have focused on the association between DcR3 and HCC, reporting increased local and systemic expression of DcR3 in patients with HCC [14–16]. On the other hand, a systematic evaluation of the levels of circulating DcR3 protein during the progression from chronic hepatitis to cirrhosis and, eventually, to HCC has not been explored so far. In addition, the potential prognostic significance of elevated serum DcR3 protein in patients with chronic liver disease is unknown at present.

We undertook the present study to comparatively evaluate the sDcR3 protein levels in patient populations at various stages of chronic liver disease (chronic hepatitis versus cirrhosis versus HCC). We report elevated levels of circulating DcR3 in these conditions, independently of the cause of liver disease, and provide evidence for a critical association between liver fibrosis severity and increased sDCR3. We also demonstrate that sDcR3 levels positively correlate with adverse disease outcomes and may therefore serve as a poor prognostic factor in patients with chronic liver disease.

## 2. Materials and Methods

### 2.1. Patients

The study population consisted of 125 consecutive patients with chronic liver disease, who were followed up at our department between January 2009 and December 2011. Chronic viral hepatitis (CVH) was diagnosed in patients without any sign of decompensated cirrhosis (DC) or HCC, who had positive hepatitis B virus (HBV) surface antigen or antibodies to hepatitis C virus (HCV) for at least 6 months and detectable viremia of HBV or HCV, respectively. DC was diagnosed in patients with any type of chronic liver disease who had a history of at least one sign of liver decompensation (ascites, variceal bleeding, hepatic encephalopathy, and nonobstructive jaundice). The diagnosis of HCC was based on histological and/or radiological findings according to international criteria [17].

Demographic and clinical information was obtained from the patients' medical records. Laboratory parameters were recorded concomitantly with the determination of sDcR3 levels. In patients with CVH who had undergone a liver biopsy, the severity of histological lesions for both necroinflammatory activity (grade) and fibrosis (stage) was assessed according to the classification of Ishak et al. [18]. All biopsies were considered to be adequate as they fulfilled the minimum requirements for adequate liver specimens (at least 6 portal tracts and length ≥2 cm). The severity of fibrosis was also assessed by shear wave elastography (SuperSonic Imagine, SA, Aix-en-Provence, France) and expressed as liver stiffness values in kPa in a subgroup of chronic liver disease patients. The severity of liver disease was assessed by the Model for End-Stage Liver Disease (MELD) in patients with DC. Further important divisions of patients' subgroups (such as treatment-naive patients in the CVH group or classification according to the presence of fibrosis) were identified and used in our analyses. In particular, patients were considered to have severe fibrosis if they had histological fibrosis stage of ≥4 at liver biopsy or liver stiffness value of >10 kPa at elastography. In addition, patients were considered to have advanced liver disease if they had severe fibrosis according to the previous definition or clinical signs of decompensated cirrhosis.

Age- and sex-matched healthy blood donors (controls) were planned to be included at a ratio of 1 per 2 cases. However, it was eventually impossible to find appropriate HC for all cases because of the old age of some patients. Thus, 48 healthy controls were eventually included. The study was approved by the local ethics committee and was in line with the Helsinki Declaration of 1975, as revised in 2008. Informed consent was obtained from all patients and controls.

### 2.2. Sample Collection

Venous blood samples were collected from patients and controls and centrifuged at 3000 rpm. In cases with elastography, a venous blood sample was collected just prior to the test. In cases with biopsy, a venous blood sample was collected on the day of admission for liver biopsy. Sera were separated, aliquoted, and stored at −80°C until being used. Ascitic fluid was also collected from a subgroup of cirrhotic patients who were admitted for therapeutic paracentesis. Ascitic fluid was collected and centrifuged at 3000 rpm. The supernatant was collected and stored immediately at −80°C.

### 2.3. Measurement of Concentration of DcR3 by ELISA

The levels of DcR3 in serum and ascites were determined using the human DcR3 DuoSet ELISA Development Kit (R&D Systems, MN), according to the manufacturer's instructions with minor modifications. Briefly, 4.0 *μ*g/mL of mouse anti-human anti-DcR3 antibody was incubated overnight in flat-bottomed microplates. Nonspecific binding was blocked with 1% BSA in PBS. Following thorough washing, 100 *μ*L of diluted sera was added and incubated overnight. After further washing, biotinylated mouse anti-human anti-DcR3 antibody was added (2.0 *μ*g/mL) and incubated for 2 h at room temperature. Finally, streptavidin-HRP (R&D Systems) was added for 30 min. After removing HRP excess, 100 *μ*L of 1 : 1 mixture of H_2_O_2_ and tetramethylbenzidine (R&D Systems) was added and absorbance was measured at 450 nm and corrected at 570 nm after 30 min. Absorbance of each sample was plotted against a standard curve produced by serial dilutions of recombinant human DcR3. Concentrations were calculated via logarithmic analysis. All samples with an absorbance of less than the average of the zero standards + 2SD were considered as nondetectable. The lower limit of detection for the assay was 0.19 ng/mL. To avoid interassay variability, in each separate assay, we run, in parallel, similar numbers of samples from patients and controls.

### 2.4. Statistical Analysis

Nonparametric tests were used for statistical analysis because of the highly skewed distribution of DcR3 levels associated with a great number of nondetectable values. In particular, comparison between groups was performed by Mann-Whitney (2 groups) or Kruskal-Wallis (>2 groups) test. Values of DcR3 in the serum and ascites from the same patient were compared by Wilcoxon test. Categorical variables were compared by chi-squared test. Spearman's *r*-test was used to assess correlations of DcR3 with other variables. Receiver operating characteristic (ROC) curve analysis was used to determine specificity, sensitivity, and optimal threshold values of sDCR3 (i.e., value offering the highest sum of sensitivity and specificity). In all cases, a *P* value of <0.05 was considered to be significant.

## 3. Results 

### 3.1. Patient Characteristics

The main patient- and disease-related characteristics are shown in Table 1. As expected, patients with CVH were younger than patients with DC and/or HCC. In the group of DC, there was an equal distribution of cases to CVH and alcoholic liver disease subgroups, whereas HCC cases were mostly related to HBV and HCV infections.

### 3.2. Serum DcR3 Protein Concentration Is Elevated in Chronic Liver Disease

A considerable proportion of controls (29%) had serum DcR3 levels below the detection limit of the assay. This was also the case in 19% of patients with CVH. In contrast, all patients with either DC or HCC had detectable sDcR3 (*P* < 0.001).

**Figure 1:**
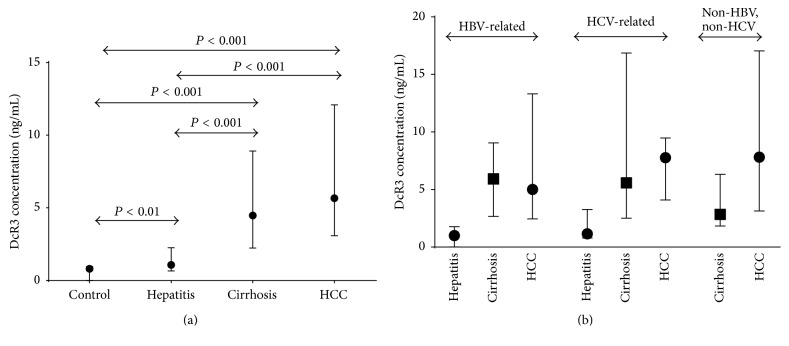
(a) DcR3 protein is highly elevated in the systemic circulation of patients with chronic liver disease. (b) sDcR3 levels are dependent upon the presence of cirrhosis and not the underlying cause of liver disease. Concentration of DcR3 in the sera was measured by ELISA. Values are expressed as median ± interquartile range. Comparison between groups was performed by Mann-Whitney test.

The levels of sDcR3 were significantly elevated in patients with chronic liver disease compared to controls (2.6 (0–63.8) versus 0.8 (0–7.9) ng/mL; median (range) *P* < 0.001) (Figure 1(a)). When separate groups were compared, we observed a gradual increase in median (range) sDcR3 levels from controls (0.8 (0–7.9) ng/mL) to 2.7-fold average elevation in CVH patients (1.1 (0–4.2) ng/mL, *P* = 0.006), an 8.8-fold increase in DC patients (4.5 (0.9–61.8) ng/mL, *P* < 0.001), and, finally, a 10.2-fold increase in patients with HCC (5.7 (0.6–63.8) ng/mL, *P* < 0.001). There was also a significant difference in sDcR3 levels between patients with CVH and those with DC (*P* < 0.001) or HCC (*P* < 0.001), while no difference was observed between patients with DC and HCC.

### 3.3. Serum DcR3 Protein Concentration Correlates with Disease Progression and Fibrosis in Patients with Chronic Liver Disease

In patients with CVH, there was no association between sDcR3 levels and age, sex, or viral genotype (for HCV-related cases). Furthermore, we observed that the concentration of sDcR3 in the serum was not associated with any specific etiology of the chronic liver disease. More specifically, when HCV and HBV infected patients were separately analyzed, the levels of sDcR3 were not associated with the underlying cause of liver disease but only with the presence of cirrhosis (Figure 1(b)). Similarly, when the subgroup of patients with cirrhosis was analyzed, no significant differences were observed among the different subgroups of patients with cirrhosis subdivided according to the etiology of liver disease (HBV, HCV, or alcohol related cirrhosis) (Figure 1(b)).

In regard to biochemical parameters, there was a trend towards higher sDcR3 values with increasing serum aminotransferases, but these correlations did not reach statistical significance (*P* = 0.085 for AST and *P* = 0.053 for ALT). In the 34 patients with a liver biopsy, we observed significant elevation of sDcR3 levels with worsening severity of fibrosis (stage). In particular, sDcR3 concentrations were higher in patients with fibrosis histological stage ≥4 than that ≤3 (median (range): 2.2 (0.6–6.8) versus 0.9 (0–5.1) ng/mL, *P* = 0.003). Similarly, sDcR3 concentrations were higher in patients with severe fibrosis than in those without severe fibrosis by either liver biopsy (histological stage ≥ 4) or elastography (stiffness > 10 kPa) (median (range): 2.2 (0–10.6) versus 1.2 (0–5.9) ng/mL, *P* < 0.001) or in those with compensated cirrhosis than in those without compensated cirrhosis (stage 5-6 or stiffness at elastography >13 kPa) (3.5 (1.8–10.6) versus 1.2 (0–6.5) ng/mL, *P* < 0.001).

A trend for higher sDcR3 values was also observed in patients with moderate or severe necroinflammatory activity (histological grade ≥ 7) than those with mild necroinflammatory activity at liver biopsy (grade ≤ 6) (1.4 (0.9–5.7) versus 0.7 (0–6.8) ng/mL, *P* = 0.096). Interestingly, when only treatment-naive patients (in the subgroup of CVH) were considered, sDcR3 concentrations were significantly higher in patients with moderate/severe inflammation than in those with mild inflammation (1.7 (0.9–3.7) versus 0.6 (0–5.0) ng/mL, *P* = 0.024).

**Figure 2:**
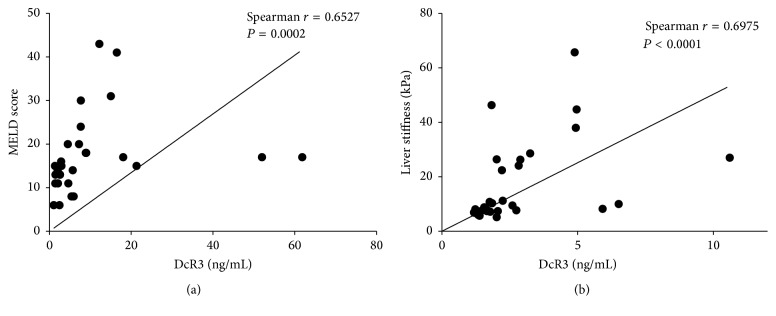
Serum concentration of DcR3 strongly correlates with markers of disease progression in patients with chronic liver disease. The serum concentration of DcR3 was measured by ELISA. Ishak scores (histological stage) were retrieved from the histological reports from patients with available liver biopsies. MELD scores were calculated for cirrhotic patients. The correlation of sDcR3 concentration with (a) the MELD score of patients with cirrhosis is shown; (b) the liver stiffness values of patients with chronic liver disease as measured by shear wave elastography. Statistical significance of correlation was calculated by use of Spearman's *r*-test.

In patients with DC, there was a positive correlation between sDcR3 levels and MELD scores (Spearman *r* = 0.653, *P* < 0.001) (Figure 2(a)). In addition, sDcR3 levels also correlated positively with other markers of severity of cirrhosis and particularly of hepatocellular synthesis, such as the international normalized ratio (*r* = 0.662, *P* < 0.001) and serum bilirubin levels (*r* = 0.609, *P* < 0.001) (Table 2). All these correlations were independent of a specific liver disease etiology.

In the subgroup of 32 patients (which included patients with all types of liver disease but not established DC cases) who had undergone shear wave elastography, a strong positive correlation between sDcR3 levels and liver stiffness measurements was found (Spearman *r* = 0.698, *P* < 0.001) (Figure 2(b)).

Finally, as we inferred from the aforementioned findings a possible association of sDcR3 with fibrosis, we used receiver operating characteristic (ROC) curve analyses in order to determine the use of sDcR3 values for diagnosis of severe fibrosis or advanced liver disease. In CVH patients without DC, the area under the ROC curve for the diagnosis of severe fibrosis (histological stage ≥ 4 or liver stiffness > 10 kPa) was 0.771 (*P* = 0.001) and the optimal cutoff value of sDcR3 levels was 1.82 ng/mL offering sensitivity of 83% and specificity of 80%. When patients with DC were also included, the area under the ROC curve for the diagnosis of advanced liver disease was 0.852 (*P* < 0.001) and the optimal cutoff value of sDcR3 levels was 1.98 ng/mL offering sensitivity of 81% and specificity of 80%.

Taken together, these results demonstrate that the presence of chronic liver disease is associated with significant elevations in circulating DcR3, with gradual increases observed with advanced stages of hepatic injury and more specifically with worsening fibrosis.

### 3.4. DcR3 Protein Is Abundantly Detected in Ascitic Fluid of Cirrhotic Patients

**Figure 3:**
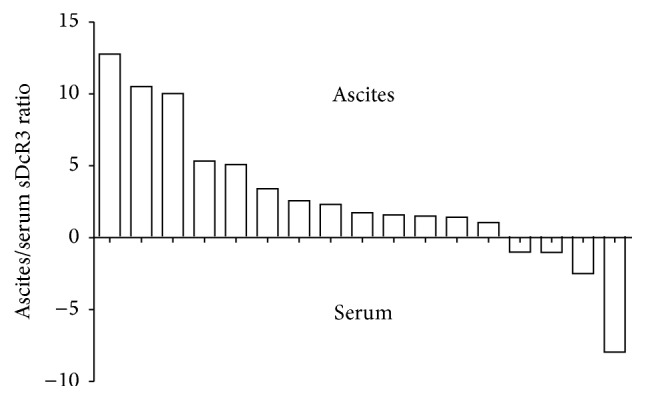
DcR3 protein is abundantly detected in ascitic fluid of cirrhotic patients. Serum and ascitic fluid were concomitantly obtained from individual patients with cirrhosis and the concentration of DcR3 was measured by ELISA in both. The ascites-to-serum ratio was determined. Each bar shown in the graph corresponds to a single individual.

DcR3 protein was always detected in ascitic fluid. To further establish a connection between DcR3 levels in ascites and liver dysfunction, we concomitantly calculated and compared the levels of sDcR3 protein in the ascitic fluid and the peripheral blood of individual patients. There was a correlation between the DcR3 levels in the ascitic fluid and the serum of patients with DC (*r* = 0.417, *P* = 0.09). In the vast majority of cases (88%), the concentration of DcR3 in ascites far exceeded the respective value in the serum (*P* = 0.013). This increase ranged from 150 to1270% (Figure 3).

## 4. Discussion

In the current study, we report that circulating DcR3 protein is elevated in patients with chronic liver disease and may serve as an indicator for disease progression, such as development of fibrosis during chronic hepatitis and functional deterioration in cirrhosis.

Several findings support this concept. First, patients with DC had significantly higher concentrations of DcR3 in comparison to patients with CVH. While it is true that patients with CVH were younger than patients with DC and/or HCC in our cohort, this difference did not have any influence on data analysis as sDcR3 concentration has been shown to not be affected by age in previous studies [10, 19]. We observed that a considerable proportion of patients in the CVH group did not have detectable DcR3 in the serum, resembling the situation in HC. In contrast, sDcR3 was always present in the serum of patients with decompensated cirrhosis. This indicates that as soon as decompensated cirrhosis is established, secretion of DcR3 becomes a stable phenotype. Second, we observed that the association between cirrhosis or increased liver stiffness in SWE and elevated DcR3 occurred irrespectively of the specific etiology of chronic liver disease. This indicates that it is not a specific causative agent (HBV, HCV, and alcohol) that drives the elevation of sDcR3, but the inflammatory/fibrotic course per se. These findings are in line with previous work by Kim et al. who reported that increased immunolocalization of DcR3 protein and detection of DcR3-specific mRNA sequences in chronic HCV-related liver disease were mainly seen in areas with prominent fibrosis [20]. Interestingly, two recent studies may support this possible role of DcR3 in the inflammatory/fibrotic pathway. It was reported that DcR3 concentrations were increased in patients with primary biliary cirrhosis in both local and systemic levels when compared to healthy controls [21], while researchers from Japan suggest that sDcR3 values may be helpful in differentiating between active and inactive chronic hepatitis B in the subgroup of hepatitis B e antigen-negative patients [22]. Furthermore, in our study, within the population of patients with chronic hepatitis, the highest DcR3 values were seen in those with advanced Ishak scores in liver biopsy (i.e., with increasing levels of fibrosis). Although the number of patients in the cirrhosis subgroup (Ishak 5-6) was small, the gradual increase in sDcR3 concentration with increasing presence of fibrosis confirms the previously shown association between DcR3 and cirrhosis. Finally, sDcR3 concentrations were observed to have a strong positive correlation with the presence of liver fibrosis as assessed by SWE liver stiffness measurements.

As DcR3 exists solely in a soluble form, it is possible that the elevated sDcR3 content shown in our study originates in regions with cirrhotic transformation of the liver. The hepatic derivation of sDcR3 is also supported by our paired examinations which showed that DcR3 values in ascitic fluid far exceeded the corresponding values in the serum of the same patient. Further supporting this hypothesis are the results from a previous study, which demonstrated that, in patients with HCC, the serum concentration of DcR3 directly correlated with the presence of paracirrhosis [14]. Interestingly, our group is the first to report expression of the DcR3 protein in the ascitic fluid obtained from patients with liver cirrhosis as previously this cytokine had been only studied in ascitic samples from patients with epithelial ovarian cancer [23].

DcR3 acts as decoy-receptor for FasL and LIGHT, blocking apoptotic signals induced by these proteins [24–26]. Thus, previous research has focused on the potential role of DcR3 in carcinogenesis in a variety of neoplastic tissues in general and more specifically in the liver. Indeed, several studies have shown increased local and systemic expression of DcR3 in patients with HCC [7, 14, 15, 27]. Moreover, DcR3 was shown to protect hepatoma cells against apoptosis induced by FasL [28]. Differently from these reports, in our present study, we comparatively examined the sDcR3 protein content along the various steps of chronic liver disease. We were able to clearly demonstrate that elevation of sDcR3 is not specific for the presence of neoplastic hepatic transformation; rather, it occurs at an earlier step, as DcR3 is already present in high levels in the systemic circulation when cirrhosis is established. Longitudinal serial measurements of sDcR3 in individual patients will be required in order to better depict the temporal association between significant DcR3 elevations and transition to cirrhosis and HCC. This might be of significant clinical importance as reliable soluble markers of disease progression and fibrosis during chronic liver disease are currently lacking.

A second present finding with potential importance is the strong positive correlation between elevated serum DcR3 and progressive liver disease. Very interestingly, such an association was evident in all patient subgroups. In particular, patients with CVH and high sDcR3 levels had increased probability for advanced liver fibrosis stage, as indicated by higher Ishak and liver stiffness scores. Similarly, in the cirrhotic population, sDcR3 positively correlated with high MELD and SWE scoring, markers of functional deterioration, and the extent of liver fibrosis, respectively. Notably, a similar association with adverse outcomes appears to take place in HCC also, as it was shown that higher local or systemic DcR3 expression in this population was associated with advanced TNM stage or increased incidence of local invasion or distant metastasis [14]. A similar positive correlation of elevated DcR3 expression with unfavorable disease course was also noted not only in other neoplasms [7, 8] but also in nonneoplastic conditions [10–13]. Taken together, these results indicate that finding a highly elevated DcR3 concentration in patients with chronic inflammatory or neoplastic conditions, including chronic liver disease, may be considered a poor prognostic factor. The biological explanation for this phenomenon is unclear but given the decoy/suppressive nature of DcR3, its excessive secretion may represent a compensatory reflex to initial upregulation of its ligands (FasL, LIGHT, and TL1A), which may occur as part of the pathogenetic disease process. Alternative explanations may involve a pro-tumor-growth result of the immunosuppressive function of DcR3. Indeed, DcR3 has been shown to favor the polarization of macrophages towards an M2 phenotype, which is characterized by decreased phagocytic activity [29]. In addition, DcR3 treated macrophages adapt to the phenotype of tumor-associated macrophages, offering therefore a survival advantage to tumor cells [30]. In all, it can be speculated that the elevated DcR3 may be pathogenetically involved in cancer progression and not merely a surrogate marker of aggressive disease course.

## 5. Conclusions

Our present findings support the hypothesis that serum DcR3 is gradually elevated during chronic liver disease and reaches its peak level after fibrosis is established. In addition, DcR3 may serve as a reliable marker of progressive disease. Further studies in larger and better selected patient populations are needed to substantiate the importance of this marker in the clinical management of patients with chronic liver conditions.

## Figures and Tables

**Table 1:** Clinical and demographic characteristics of patients.

	Chronic hepatitis (*n* = 58)	Cirrhosis (*n* = 46)	HCC (*n* = 21)
Age, years (mean ± SD (range))	43.9 ± 12.7 (25–68)	64.9 ± 11.0 (46–89)	61.3 ± 9.9 (50–77)

Male sex (%)	40 (69%)	35 (76.1%)	20 (95.2%)

Cause of liver disease (*n*, %)			
HBV infection	28 (48.3%)	11 (23.9%)	9 (42.9%)
HCV infection	30 (51.7%)	14 (30.4%)	5 (23.8%)
Alcohol		14 (30.4%)	
Unknown		7 (15.2%)	7 (33.3%)

Histological grade (*n* = 34)			
Mild necroinflammation (1–6)	23 (67.6%)	NA	NA
Moderate (7–12)	9 (26.5%)	NA	NA
Severe (13–18)	2 (7.9%)	NA	NA

Histological stage (*n* = 34)			
No fibrosis (0)	14 (41.2%)	NA	NA
Minimal (1-2)	9 (26.5%)	NA	NA
Moderate (3-4)	7 (20.5%)	NA	NA
Severe (5-6)	4 (11.8%)	NA	NA

MELD score (mean ± SD (range))	NA	15.89 ± 9.27 (3–43)	

Liver stiffness (kPa) (mean ± SD (range))	7.91 ± 1.68 (5–11)	33.11 ± 13.22 (22–66)	NA

NA: not applicable.

**Table 2:** Correlation of sDcR3 with patient characteristics.

	Spearman correlation coefficient (*r*)	*P*
Chronic viral hepatitis		
Age	0.046	NS
Sex		NS
HCV genotype		NS
AST	0.331	0.085
ALT	0.369	0.053

DC		
Age	0.054	NS
Sex		NS
MELD score	0.653	<0.001
AST	0.299	NS
ALT	0.127	NS
Bilirubin	0.609	<0.001
INR	0.662	<0.001
Creatinine	0.243	NS

NS: nonsignificant value.
